# Silly walks: An urgent practical solution to the inactivity and obesity pandemics

**DOI:** 10.1113/EP093233

**Published:** 2025-11-22

**Authors:** Glenn A. Gaesser, Siddhartha S. Angadi, David C. Poole

**Affiliations:** ^1^ College of Health Solutions Arizona State University Phoenix Arizona USA; ^2^ Department of Kinesiology, School of Education and Human Development University of Virginia Charlottesville Virginia USA; ^3^ School of Health Sciences and Department of Anatomy and Physiology Kansas State University Manhattan Kansas USA

**Keywords:** cardiorespiratory fitness, exercise, Monty Python, obesity, silly walk, maximal oxygen uptake

## Abstract

To assess whether the increased energy cost of silly walking (SW) could enhance physical activity, reduce obesity and extend health span, we retrospectively analysed data from 13 healthy adults (seven males, six females; age 22–71 years) who performed three walking trials. Oxygen uptake (${\dot{V}}_{O_{2}}$; in millilitres of O_2_ per kilogram per minute), energy expenditure (EE; in kilocalories per minute) and cost per distance (in joules per kilogram per metre) were measured via expired ventilation and gas exchange. Trials included normal walking and two SWs, replicating Monty Python's Michael Palin and John Cleese in the Ministry of Silly Walks sketch (1971). Although both Cleese and Palin SWs evinced greater cost per distance, only the Cleese SW elevated ${\dot{V}}_{O_{2}}$ compared with normal walking (28 ± 5 vs. 11 ± 3 mL/kg/min; EE, +8.0 ± 2.3 and +5.2 ± 0.8 kcal/min in males and females, respectively). Replacing 1–3 min/day of normal walking with the Cleese SW would require an extra energy expenditure of 12 kcal/day (∼4400 kcal/year), approximately twice the energy equivalent of excess weight gain sufficient to explain the obesity pandemic. The EE associated with SW, requiring no additional physical activity, could potentially eliminate future weight gain and, with a modest extra investment of merely 6–23 min/day, SW could redress the obesity pandemic entirely. Crucially, increasing physical activity by SW is expected to elevate cardiorespiratory fitness (${\dot{V}}_{O_{2}\max}$), thereby diminishing inactivity‐related diseases considerably. Combined with weight loss, relative ${\dot{V}}_{O_{2}\max}$ gains could reach ∼90%. If SW had been adopted in the 1970s, the global obesity crisis might have been prevented, or at least greatly attenuated, along with commensurate improvements in fitness and health span.

## Abstract (highlights)

- **What is the central question of this study?**
  Can flailing one's limbs about in a gloriously inefficient and utterly daft manner, known as silly walking, prevent obesity, improve cardiorespiratory fitness and save humanity? Can silliness equal fitness and health?
- **What is the main finding and its importance?**
  Replacing a few minutes of one's daily trudge with a Cleese‐style, knees‐to‐the‐heavens silly walk can burn 2.5 times as much energy as a normal walk. This means that with a few minutes of silly walking per day, the global obesity pandemic could have been avoided, fitness restored, and the Ministry of Health Span could have been retired. In short, silly might very well be the sanest solution!

## 1 INTRODUCTION

### 1.1 Comedy is the lie that teaches us the truth

**FIGURE 1:**
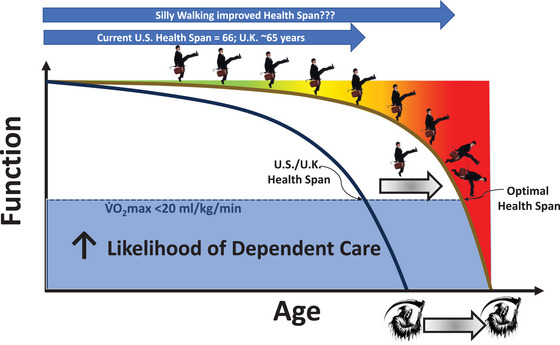
Schematic diagram depicting the current US/UK health span, which, at ∼66 years, lags behind that of most developed countries and sentences the majority of Americans and Britons to well over a decade of morbidity, presaging the likelihood of dependent care when the maximal oxygen uptake (${\dot{V}}_{O_{2}\max}$) falls below ∼20 mL/kg/min (black curve). This article explores the potential for Cleese silly walking to counter inactivity‐related disease, increase cardiorespiratory fitness and reduce body mass, thereby extending the health span (grey arrow, brown curve) such that the period of morbidity is reduced to a few months or years immediately preceding death. Adapted from Gaesser et al. (2025). See main text for more details.

Over the last century, one cardinal achievement of humanity has been the three‐decade extension of the average lifespan from birth (Olshansky, 2018). Tragically, in a failure of success, this has extended the time spent enduring chronic disease(s) such that, following a health span of ∼66 years, the latter 15‐20 years of life is spent in aged morbidity (Elliot et al., 2020; Ferucci et al., 1999; Gruenberg, 1977; Olshansky, 2018; reviewed by Gaesser et al. 2025). A Panglossian scenario, termed the ‘ideal survivorship curve’ (Fries, 1980), would entail the vast majority of our extended lifespan being health span, with a foreshortened period of months or very few years of morbidity immediately preceding death; that is, a sharp cliff of decreasing function and quality of life (Booth & Hawley, 2015; Fries et al., 2011; Lazarus & Harridge, 2018; Figure 1). Although caloric restriction shows promise, the most effective therapeutic countermeasure available to extend the health span is physical exercise (Booth & Hawley, 2015; Gaesser et al. 2025; Harber et al., 2017; Joseph & Pojednic, 2023; Kraus et al., 2019).

**FIGURE 2:**
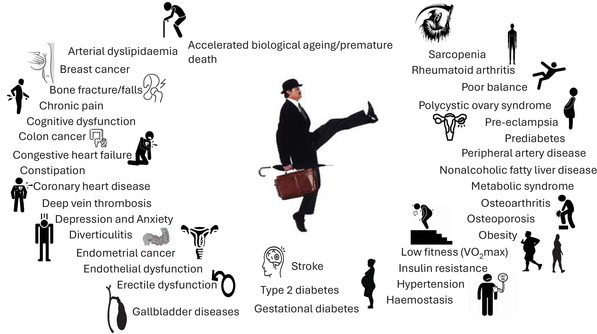
Array of pathological and clinical conditions offset/countered by physical activity (compiled from Booth et al., 2012).

Major contributing factors to the truncated health span are inactivity (Figure 2) and obesity (now classed as a disease in itself); the latter is likely to be a proxy, in most, but not all, cases, for a host of dietary and lifestyle behaviours. The prevalence of obesity has doubled in >70 countries over the past few decades (GBD 2015 Obesity Collaborators et al., 2017), with that in Europe and the USA tripling (Hales et al., 2020; Hruby & Hu, 2015; Pineda et al., 2018). This unfortunate situation constitutes the perfect metabolic storm of ready access to (often) delicious, cheap food with high caloric density (increasing calories in) conflated with a Darwinian neurophysiology and psychology choosing or modifying behaviours to minimize perceived effort (reducing calories expended), termed the theory of effort minimization in physical activity (TEMPA) (Cheval & Boisgontier, 2021).

To counter TEMPA, we propose practice of effort maximization in physical activity (PEMPA). We adopted this initiative from the heretofore unrecognized scientific genius of John Cleese and Monty Python's Ministry of Silly Walks (MoSW; https://www.youtube.com/watch?v=TNeeovY4qNU) to demonstrate that a few brief bouts per day of John Cleese‐style silly walking (SW) would be sufficient to meet the guidelines for vigorous‐intensity physical activity (Gaesser et al., 2022a, 2022b). Herein, we retrospectively explore whether silly walking, had it been widely adopted and continuously practised after the MoSW skit first aired in the early 1970s, could have prevented, or at least greatly attenuated, the obesity pandemic of the past ∼50 years.

**FIGURE 3:**
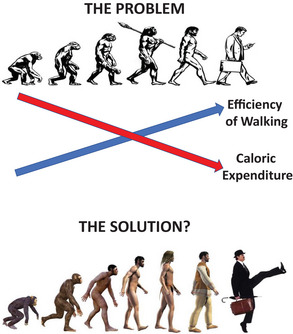
Top, the problem: Walking has become far more efficient, with a far lower calorie expenditure, as humans have evolved. Bottom: In 1970, Monty Python's Ministry of Silly Walks comedic skit presented a putative solution to counter the increased efficiency of walking. On the right is the actor and comedian John Cleese as Mr Teabag, demonstrating his iconic silly walk. From Gaesser et al. (2025).

SW strategies target solely the latter term in the caloric balance equation, elevating calories expended. In opposing TEMPA, we recognize that evolution has adapted human anatomy, biomechanics (Lai et al., 2016; Stearne et al., 2016) and physiology (Bramble & Lieberman, 2004; Cheval & Boisgontier, 2021; Lieberman, 2015) from apes such that contemporary walking is 100%–200% more efficient in *Homo sapiens* versus chimpanzees (*Pan troglodytes*) (Sokol et al., 2007; Pontzer, 2017; Figure 3). Notably, obesity is disappearingly rare in *P. troglodytes*. Moreover, given that obese humans regulate their walking pace to minimize the energetic cost such that it is surprisingly low and similar to their lean counterparts (Browning & Kram, 2005; Oliveira et al., 2020), their obesity might be intractable to the prescription of regular (normal) walking.

Before settling upon the MoSW, we contemplated other strategies for PEMPA, including walking on all fours and walking backwards. However, chimpanzees and capuchin monkeys expend the same amount of energy whether they run on two or four legs (Taylor & Rowntree, 1973), negating the utility of this strategy for humans. Although for humans at least naïve novel walking on all fours is likely to be less efficient than for these apes and monkeys, its lack of silliness will decrease collateral physiological and public health benefits. Alternatively, backward walking increases energy expenditure compared with forward walking (Chaloupka et al., 1997; Flynn et al., 1994), but has obvious limitations for reasons of safety and sociability.

Opponents of the suggested MoSW approach might rightfully point out that human evolution has promoted TEMPA via neuroscience (Prevost et al., 2010), evolutionary biology (Alexander, 1996) and biomechanics (Abram et al., 2019). Thus, TEMPA strategy, by allowing allocation of limited energetic resources to reproduction/repair (Gibson & Mace, 2006; Pontzer et al., 2016), has facilitated the proliferation of humans (∼8 billion) versus chimpanzees (perhaps 150 000–250 000) and, therefore, is the way to go.

But the humble sea squirt, *Clavelina moluccensis*, offers a peek into one frightening potential future for the human race if PEMPA, in line with MoSW principles, is not adopted. *Clavelina moluccensis* begins life much like a tadpole, with a muscular tail, backbone, brain and an eye, happily swimming around after food. But when it locates a convenient rock where the current delivers that food, it affixes to said rock and promptly absorbs its brain, backbone and muscles to become simply a mouth and digestive system (Turker, 2021). Far too many humans in contemporary Western cultures affix themselves to their couch and gorge on an electronically summoned stream of high‐calorie foods. Their muscles atrophy from lack of use, the television promotes cognitive dysfunction, and their fat stores hypertrophy. A future not unlike the humans on the Axiom spaceship in the Pixar blockbuster Wall‐E seems within the realm of possibility (https://www.youtube.com/watch?v=h1BQPV‐iCkU).

Is this our grim future? It does not have to be. The SWs of Monty Python members John Cleese and Michael Palin have been analysed biomechanically and, on the basis of gait variability scores, John Cleese's SW was 6.7 times more variable (sillier) than normal walking, versus 3.3 times for Michael Palin's SW (Butler & Dominy, 2020). Energy expenditure was not measured in that study and thus, before Gaesser et al. (2022a, 2022b), no study, to our knowledge, had determined the SW energy cost across the half‐century since the MoSW skit first aired. The novelty of this focused study is to use extant data (Gaesser et al., 2022a, 2022b) to explore the potential of SW strategies to redress the inactivity and obesity drivers of the foreshortened health span crippling modern society.

## 2 MATERIALS AND METHODS

### 2.1 Ethical approval

This study was approved by the Arizona State University Institutional Review Board (IRB number STUDY00008658) and conformed to the ethical standards of the *Declaration of Helsinki*. All participants provided written consent.

For purposes of the present focused review, we performed a secondary data analysis of the study by Gaesser et al., 2022a, 2022b), which included 13 healthy adults [six females and seven males; mean ± SD age = 34.2 ± 16.1 years; weight = 78.2 ± 22.5 kg; body mass index (BMI) = 25.6 ± 6.0 kg/m^2^]. In brief, after each participant was shown a video of the MoSW skit they performed three 5 min walking trials: one of normal gait and pace and two recreating the walks performed by John Cleese, including the major components of the silly walk performed both outdoors (whilst walking to work) and indoors (mainly in his office at the MoSW), and Michael Palin to the best of their ability. Whilst performing these trials, participants were fitted with a lightweight, portable metabolic measurement system (Carefusion, San Diego, CA, USA) previously validated against the Douglas bag method (Rosdahl et al., 2010). The distance covered was recorded, and the average walking speed was calculated for each walk. Ventilation and gas exchange were measured throughout each trial for determination of oxygen uptake (${\dot{V}}_{O_{2}}$; in millilitres of O_2_ per kilogram per minute) and carbon dioxide production, which were used to compute energy expenditure as described previously (Gaesser et al., 2022a, 2022b). With α set at *p* < 0.05, differences in energy expenditure (EE; in kilocalories per kilogram per minute) amongst the three walks were determined using linear mixed models with appropriate Bonferroni corrections for multiple comparisons. These values were then analysed for the express purpose of estimating the potential of SW to provide an exercise stimulus sufficient to alter the caloric balance to redress the obesity pandemic.

To determine the mean increase in body weight during the decades of increasing obesity prevalence, we used data from the National Health and Nutrition Examination Surveys (Fryar et al., 2021, 2018). To calculate the energy content of the additional body weight gained, we assumed that 100% of the additional weight was fat and that the energy equivalent of 1 kg of body fat is ∼7700 kcal (Heymsfield et al., 2012; Wishnofsky, 1958). To estimate the amount of SW necessary to offset the additional weight gain that occurred during the decades of increasing obesity prevalence, we relied on the data of Hill et al. (2009), who proposed that the energy gap for weight gain prevention be estimated as twice the daily energy accumulation.

## 3 RESULTS

**FIGURE 4:**
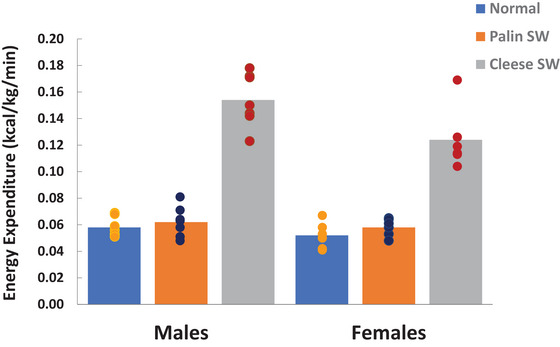
Energy expenditure for males and females walking normally (blue) and engaging in the Palin (orange) or Cleese (grey) silly walk (SW). Data from Gaesser et al. (2022a, 2022b).

**FIGURE 5:**
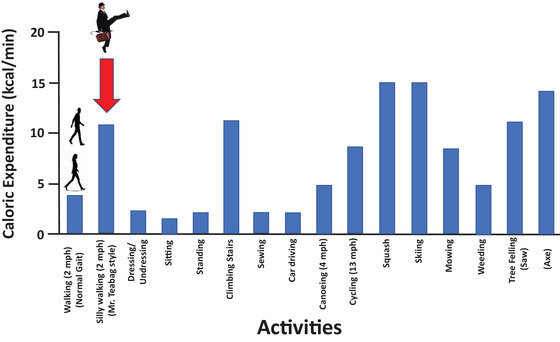
Caloric expenditure for normal and Cleese silly walking at 2 m.p.h. from Gaesser et al. (2022a, 2022b) compared with published values for various sports and activities (Astrand & Rodahl, 1986: p. 505).

**FIGURE 6:**
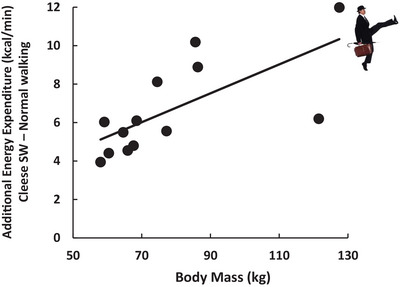
Association between additional energy expenditure (in kilocalories per minute; 1 kcal = 4.18 kJ) and body mass (in kilograms) for participants’ Mr Teabag (i.e., Cleese) silly walking (SW). Pearson correlation: *r* = 0.69, *p *= 0.008 (data from Gaesser et al., 2022a, 2022b).

Although both the Cleese SW and Palin SW increased the energy cost of walking per unit distance (normal = 3.6 ± 0.4 J/kg/m; Palin SW = 8.9 ± 1.7 J/kg/m; Cleese SW = 11.2 + 2.8 J/kg/m), only the Cleese SW significantly elevated EE per unit time (Figure 4). The Cleese SW elevated EE ∼2.5‐fold in comparison to normal walking, achieving a mean ${\dot{V}}_{O_{2}}$ of 28 ± 5 mL O_2_/kg/min [∼8 resting metabolic equivalents (METs); 1 MET = 3.5 mL O_2_/kg/min]. For male participants (64.6–127.5 kg), the greater EE ranged between 5.5 and 12.0 kcal/min during the Cleese SW. For females (58.0–121.5 kg), the greater EE ranged between 3.9 and 6.2 kcal/min. Figure 5 compares the average gross EE of Cleese SW (11.0 ± 3.5 kcal/min) with normal walking (4.3 ± 1.4 kcal/min) and with a range of daily activities and sporting pursuits. Part of the genius of Cleese SW, in part, is that, because SW involves considerable work against gravity, EE scales against body mass (Figure 6). Accordingly, caloric expenditure increases more steeply as a function of body mass, potentiating the benefit of SW to redress even extreme cases of obesity.

## 4 DISCUSSION

The ∼2.5‐fold higher ${\dot{V}}_{O_{2}}$ (and EE) when performing the Cleese SW in comparison to normal walking would clearly constitute heavy‐ or severe‐intensity exercise depending on the exact movement characteristics and fitness of the individual. Note that this EE might not achieve the levels possible with squash, skiing or axing trees, but it exceeds that of many other activities and domestic chores and is similar to climbing stairs (but so much more creative and entertaining!) (Figure 5). Owing to its vigorous intensity (∼8.7 METs for males; ∼7.0 METs for females), 11 min of SW per day would meet current physical activity guidelines (Garber et al., 2011), which we have highlighted previously (Gaesser et al., 2022a, 2022b). Even if accrued in short bouts of 1–2 min at a time, the positive health impact of silly walking could be substantial because cardiovascular benefits are virtually the same regardless of whether a given amount of exercise is performed in a single session or in several short bouts throughout the day (Garber et al., 2011).

The following discussion examines (in the context of energy balance) the amount of SW necessary to have prevented the obesity pandemic of the past ∼50 years. These are not mere dulcet platitudes delivered to a world overly saturated with get fit‐ and get thin‐quick schemes that overpromise and underdeliver. Moreover, without the dangers of yo‐yo dieting (Gaesser et al. 2025), harmful side effects of pharmaceuticals or expensive gym memberships, we substantiate the pressing potential for SW to extend the health span.

### 4.1 Obesity pandemic

The global obesity pandemic is a result of small imbalances in energy intake and EE occurring progressively over time. In the past five decades, BMI globally has increased by ∼2.5 kg/m^2^ [NCD Risk Factor Collaboration (NCD‐RisC), 2016] and by greater amounts in Western societies. For example, based on National Health and Nutrition Examination Survey (NHANES) data in the USA, the mean BMI of men and women increased by 3.8 and 4.5 kg/m^2^, respectively, between 1976 and 2018 (Fryar et al., 2021, 2018). Consequently, during that ∼40 year period the body weight of the average US adult (both men and women) increased by ∼11.5 kg (∼25 lb).

Presuming all of the 11.5 kg weight gain is body fat and that 1 kg of adipose tissue contains ∼7700 kcal (Wishnofsky, 1958; but see Heymsfield et al., 2012), the accumulated energy content approximates 88 550 kcal. This corresponds to an energy surplus of ∼2200 kcal/year or ∼6 kcal/day. Hill et al. (2009) proposed that the energy gap for prevention of weight gain be estimated as twice the daily energy accumulation, which takes into consideration that excess energy is not stored with 100% efficiency (Bray & Bouchard, 2020). Thus, from a purely EE perspective, the accumulated body fat on the average US adult since the late 1970s might have been prevented with as little as 1–3 min/day of silly walking. For our participants, the additional energy expenditure during silly walking compared with normal walking ranged from 5.5 to 12.0 kcal/min for males (i.e., ∼1–2 min/day to achieve the 12 kcal/day energy gap) and from 3.9 to 6.2 kcal/min for females (i.e., ∼2–3 min/day to achieve the 12 kcal/day energy gap). Even with our small sample size, our results suggest that for most adults, the amount of silly walking required to achieve the necessary EE to prevent weight gain is <3 min/day.

The NHANES data likewise suggest a very small energy gap, ∼12 kcal/day, to explain the difference in body weight of US adults over that time period when obesity prevalence tripled. For other countries, the energy gap has been estimated to range between 8 and 45 kcal/day (Hill et al., 2009). Thus, the amount of silly walking necessary to prevent weight gain will vary, but probably would not exceed ∼10 min/day for most people. Physical activity bouts of <10 min duration were found to be related to lower BMI and obesity risk amongst adults in the NHANES 2003–2006 cohort (Fan et al., 2013). In fact, each 1 min of higher‐intensity physical activity achieved in short bouts lasting <10 min was associated with lower BMI compared with each 1 min of similar‐intensity physical activity achieved in bouts lasting >10 min. These findings support the recommendation of brief bouts of silly walking lasting no more than ∼2–3 min, interspersed throughout the day, for weight control and obesity prevention.

The added advantage of the short‐bout approach is that it is less likely to trigger a TEMPA override that would act to limit higher‐intensity physical activity. Perceived effort is both intensity and time dependent, and we have reported previously that high‐intensity intervals lasting 1–2 min elicit a rating of perceived exertion of only ∼5–6 on the 10‐point modified Borg scale (Noble et al., 1983; Tucker et al., 2015). This is particularly relevant to the Cleese silly walk because our participants averaged 28 mL/kg/min whilst performing the Cleese silly walk, corresponding to ∼8 METs (range 5.5–10.0 METs).

Many adults will downregulate time spent in spontaneous physical activity when increasing energy expenditure by structured exercise (Pontzer et al., 2016). However, we have previously advocated for an SW approach that does not add to physical activity time commitments but, rather, replaces a low‐EE activity (normal walking) with a high‐EE activity (silly walking) (Gaesser et al., 2022a, 2022b).

Body mass is also likely to influence the amount of SW necessary to achieve the energy gap. The additional EE of the Cleese SW (in excess of normal walking) was positively correlated with body mass (*r* = 0.69; *p* = 0.008), with the heavier individuals reaping greater energetic benefit (Figure 6)! This could be useful for preventing future weight gain. Men aged 18–24 years in the UK are predicted to gain an average of 9.4 kg (20.7 lb) over the next decade (Katsoulis et al., 2021). The amount of SW necessary to prevent this weight gain is likely to be similar to or slightly less than that described above for US adults in NHANES cohorts between 1976 and 2018. Thus, SW could prevent almost one‐third of men in the normal‐weight BMI category from transitioning to overweight and a similar portion of overweight individuals from transitioning to obese over 10 years (Katsoulis et al., 2021). Women would experience a compatible directional result.

Whether silly walking could reverse the obesity pandemic via weight loss is highly speculative, as suggested by the constrained total energy expenditure model of physical activity in adult humans (Pontzer et al., 2016). That model posits that the total energy output by the body is homeostatically controlled within a narrow range and does not increase commensurately with physical activity. However, that model pertains to ‘normal’ physical activity. A silly walking intervention has never been tested, and it is possible that silly walking represents such a novel form of ambulatory activity that heretofore studied models of human locomotion do not apply. Moreover, even in studies that have documented large discrepancies between expected and observed weight loss in response to exercise training (Manthou et al., 2010; Sawyer et al., 2015), there is considerable heterogeneity in individual responses, with a significant percentage of responders (or super‐responders) that achieves a reduction in total body energy content equal to or greater than the cumulative EE of all exercise sessions. On the purely optimistic assumption that SW would produce 100% responders, we have taken the liberty to speculate on how much SW would be necessary to normalize BMI and thus reverse the obesity pandemic.

**FIGURE 7:**
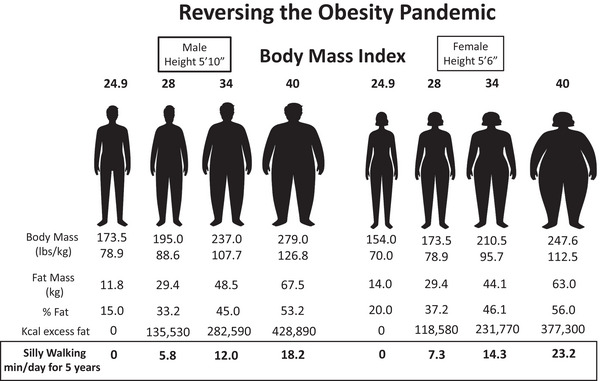
Calculations of minutes per day of Cleese silly walking necessary to reverse the obesity pandemic. Considered as losing sufficient fat mass (at 7700 kcal/kg) to achieve/return to a body mass index of 24.9 kg/m^2^ from body mass indexes of 28, 34 and 40 kg/m^2^, which covers a continuum encapsulating the vast majority of overweight and obese individuals.

Considering that each kilogram of body fat represents 7700 kcal (see above), Figure 7 calculates the minutes of Cleese SW per day over 5 years required to re‐establish a ‘normal’ BMI of 24.9 kg/m^2^ for men and women from BMIs of ≤40 kg/m^2^; assuming no increase in caloric consumption over that period and no downregulation of all sources of total daily EE outside of the EE associated with SW. The latter assumption is feasible because, as described above, SW might not elicit a TEMPA override to constrain total daily energy expenditure. Energy intake amongst adults generally decreases with age (Giezenaar et al., 2016), and the effect of exercise on energy intake is characterized by significant individual heterogeneity (Blundell & Beaulieu, 2023). Note the exceptionally modest time investment. The daily requirement of SW (∼6–23 min/day) assumes an average EE of 12.9 kcal/min for males and 8.9 kcal/min for females (based on our participants) and assumes that the SW would be in addition to normal physical activity. If the SW is in lieu of normal walking, that is, replacing normal walking with SW so as not to add to the total amount of daily walking as we have advocated previously (Gaesser et al., 2022a, 2022b, 2025) and above, the daily minutes required for fat loss would be ∼35% higher than those presented in Figure 7.

The elevated metabolic requirements of the Cleese silly walk are expected to increase cardiorespiratory fitness, although this needs to be tested rigorously. The average ${\dot{V}}_{O_{2}}$ during the Cleese silly walk was 30 mL O_2_/kg/min (∼8.7 METs) for our male participants and 25 mL O_2_/kg/min (∼7.1 METs) for our female participants. Also, it is relevant that heavy‐/severe‐intensity exercise recruits a greater proportion of less efficient higher‐order fast‐twitch fibres (type IIa/IIx) and also incurs a substantial ${\dot{V}}_{O_{2}}$ slow component (Poole, 1994). Thus, not only will bouts >3 min or so in length generate this additional metabolic cost but they might also specifically benefit aged and patient populations who exhibit greater reliance on these fibre types (Poole et al., 2012). This vigorous‐intensity exercise for adults will increase cardiorespiratory fitness (Garber et al., 2011), with 75 min/week (∼11 min/day) of such exercise meeting public health guidelines for physical activity and reducing risk of all‐cause mortality (Kraus et al., 2019). Furthermore, should participants wish to increase the speed and intensity of their SW above that tested herein, it has been demonstrated that ∼20% increases in ${\dot{V}}_{O_{2}\max}$ can be achieved in 4 weeks with as little as 60 min/week of severe‐ or 105 min/week of heavy‐intensity training (Poole & Gaesser, 1985). For those especially time‐strapped individuals, Bailey et al. (2009) had participants achieve a 7% increase in ${\dot{V}}_{O_{2}\max}$ over 2 weeks with <10 min/week of all‐out cycle ergometer Wingate tests. Thus, depending on the motivation and abilities of individuals to SW intensively, there is enormous potential for beneficial cardiorespiratory improvements (and thus health span extension) with a surprisingly minimal time investment.

**FIGURE 8:**
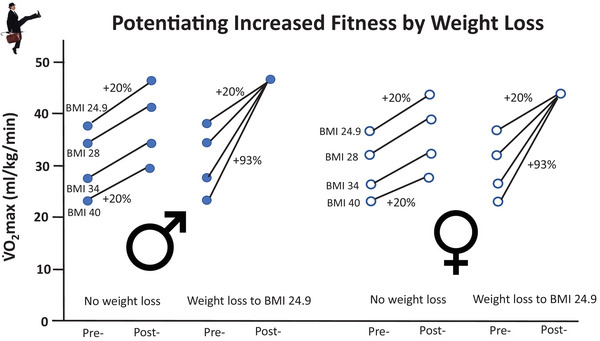
Demonstration of the average increase in cardiorespiratory fitness (${\dot{V}}_{O_{2}\max}$) expected from exercise training in the heavy/severe intensity domains and how this is potentiated by weight loss [decreasing body mass index (BMI) to 24.9 kg/m^2^] when considering relative ${\dot{V}}_{O_{2}\max}$ (i.e., millilitres per kilogram per minute). Exemplars here are for individuals with an initial 3.0 L/min (male) or 2.5 L/min (female) ${\dot{V}}_{O_{2}\max}$, who increase their absolute ${\dot{V}}_{O_{2}\max}$ by 20% after silly walking and restore their BMI of 24.9 kg/m^2^ after 5 years of silly walking. Ignoring the ∼1% expected decline in ${\dot{V}}_{O_{2}\max}$ with advancing age. See main text for more details.

Even without consideration of an exercise‐induced increase in ${\dot{V}}_{O_{2}\max}$, weight loss alone could potentially have a substantial impact on aerobic fitness. Taking an extreme exemplar from Figure 7, for an individual with a BMI of 40 kg/m^2^ who engages in ∼18–23 min/day of Cleese SW, reducing BMI to 24.9 kg/m^2^ (loss of ∼115 lb for men and ∼94 lb for women; Figure 8) will elevate relative ${\dot{V}}_{O_{2}\max}$ by a prodigious 93%! This elevated fitness in and of itself would yield substantive public health benefits, because physical activity health improvements are independent of BMI status and weight loss (Gaesser & Angadi, 2021). In fact, aerobically fit adults with obesity have lower all‐cause mortality risks than unfit non‐obese adults (Gaesser et al., 2015; Weeldreyer et al., 2025). For this reason alone, the SW initiative will have massive benefits for health and health span.

### 4.2 Reducing health‐care costs

SW cannot help but reduce health‐care costs. Worldwide, obesity will consume 3.6% of gross domestic product by 2060, doubling the current amount and representing a substantial wastage of national resources (Okunogbe et al., 2021). In the UK, the National Health Service medical costs of obesity in 2014–2015 were ₤6.1 billion ($7.6 billion), with that to society at large estimated to be ∼₤27 billion ($33.8 billion) (Health Matters, 2017). These are calculated to rise to ₤9.7 and ₤49.9 billion, respectively, by 2050, hence there is no time to lose. This makes the ₤248 million funding claimed by Cleese for the MoSW in the 1970s seem like a real bargain even when adjusted for the interim inflation (₤3.4 billion). The lower health‐care costs will be realized with universal adoption of SW, regardless of its impact on obesity. For instance, in the USA, annual health‐care costs for aerobically fit obese men were ∼$10 000–$27 000 lower than costs for unfit men in the normal BMI category (de Souza de Silva et al., 2019). Moreover, increasing fitness (Bailey et al., 2009; Gaesser & Angadi, 2021; Poole & Gaesser, 1985) and simultaneously decreasing obesity (see Figure 8) would potentiate the health and predicted health span benefits (Blair et al., 1989; Butler, 1977; Gaesser et al., 2015).

SW will improve the health of both silly and non‐silly walkers. Use of the PEMPA model, based on MoSW principles, has significant public health implications for both the silly and the non‐silly walkers. SW is likely (at least initially) to evoke hilarious laughter amongst non‐silly walkers. This will also confer significant health benefits on them, because laughter has beneficial effects on blood pressure (Sugawara et al., 2010), vascular function (Miller et al., 2006; Sugawara et al., 2010), arterial stiffness (Sugawara et al., 2010) and diabetic complications (Noureldein & Eid, 2018) Moreover, laughter improves pain tolerance (Lapierre et al., 2019) and cardioverts arrhythmias (Pallas & Smiles, 2019), with frequent laughter reducing cardiovascular disease risk (Hayashi et al., 2016). What other public health intervention, except perhaps quitting smoking, can boast such splendid healthful effects in users and non‐users? We recommend urgent adoption of SW as a public health measure to combat the twin pandemics of obesity and inactivity‐related diseases in silly and non‐silly walkers (Figure 2).

Although EE for the Palin SW was not different from that of normal walking, it should not be dismissed as pointless for preventing excess weight gain. The energy cost per unit distance for the Palin SW (8.9 ± 1.7 J/kg/m) is only slightly lower than the Cleese SW (11.2 ± 2.8 J/kg/m) and is 2.5 times greater than that of normal walking (3.6 ± 0.4 J/kg/m) because of its slow speed, at 1.76 km/h (1.1 mph). Although this SW mode might not be acceptable for those with significant time constraints (or negotiating a crosswalk through a busy intersection!), the Palin SW might be preferred by individuals without time constraints, such as retired persons. Despite its lack of silliness (according to Mr Teabag, the character played by John Cleese, ‘the right leg isn't silly at all, and the left leg merely does a forward aerial half‐turn every alternative step’), the higher cost of movement per distance covered would increase EE without necessitating the higher ${\dot{V}}_{O_{2}}$ required of the Cleese SW. The tangible benefits include the higher energy cost of the Palin SW (per unit distance) in addition to the laughter they might enjoy watching their fitter and more agile children and grandchildren perform the more demanding elements of the Cleese SW. It would be a win–win.

### 4.3 Limitations and considerations for future investigation

At the present time, we cannot advocate extension of the SW initiative to other forms of exercise, such as swimming or cycling. Not only are they largely body mass independent for EE during activity, but we note that a short period of silly swimming usually precedes death by drowning, much as silly cycling precedes death by catastrophic vehicular impact. ‘Groucho running’ or actually ‘Groucho walking’, because it lacks an aerial phase, has been shown to increase EE in comparison to normal running (McMahon et al., 1987). Although the absolute increase in EE of Groucho running relative to normal running is similar to that of the Cleese SW compared with normal walking, Groucho running has only been studied in well‐trained endurance runners at speeds that require an EE beyond the aerobic capacity of most adults. Thus, SW as studied herein, which may include crouched steps, as in the Cleese SW, might have more universal relevance and appeal.

SW constitutes vigorous‐intensity exercise, which, for other forms of exercise, such as running, can evoke the neuroendocrine phenomenon known as ‘runner's high’, motivating some individuals to sustain exercise (Hausenblas et al., 2017; Raichlen et al., 2012). Whether SW will generate a ‘walker's high’ that normal walking cannot is an important question that remains to be investigated. These limitations could be overcome with immediate MoSW grant support.

## 5 CONCLUSIONS

Half a century ago, and sadly ignored by medical professionals, Monty Python's iconic SW skit provided a powerful exercise programme that would have increased the health span. For adults worldwide, and especially in developed countries with a limited health span who endure decades of morbidity towards the end of life, irrespective of BMI, a few minutes a day dedicated to SW will abrogate the risk of inactivity‐related diseases. Moreover, the data analysis herein clearly supports that, had the MoSW initiative been adopted in the early 1970s, the entire global inactivity and obesity pandemics might have been prevented. That opportunity was sadly missed. However, we also demonstrate a plan for SW to reverse the twin pandemics of inactivity and obesity and implore that it is NEVER too late to start!

## AUTHOR CONTRIBUTIONS

Conceived and designed the work: Glenn A. Gaesser, Siddhartha S. Angadi and David C. Poole. Gathered the data: Glenn A. Gaesser. Contributed to data analysis and interpretation: Glenn A. Gaesser, Siddhartha S. Angadi and David C. Poole. Drafted the manuscript and each revised it for critical intellectual content: Glenn A. Gaesser, Siddhartha S. Angadi and David C. Poole. All authors approved the final version of the manuscript and agree to be responsible for all aspects of the work in ensuring that questions related to the accuracy or integrity of any part of the work are appropriately investigated and resolved. All persons designated as authors qualify for authorship, and all those who qualify for authorship are listed.

## CONFLICT OF INTEREST

None declared.
